# Cardiopulmonary Bypass Circuit Modification Proposal for Modified Ultrafiltration in Children

**DOI:** 10.21470/1678-9741-2025-0028

**Published:** 2025-08-22

**Authors:** Amanda Vargas Scaranaro, Rafael da Silva Policarpo, Ulisses Alexandre Croti, Renata Geron Finoti

**Affiliations:** 1 CardioPedBrasil®, Centro do Coração da Criança, Hospital da Criança e Maternidade de São José do Rio Preto (FUNFARME), Faculdade de Medicina de São José do Rio Preto (FAMERP), São José do Rio Preto, São Paulo, Brazil

**Keywords:** Extracorporeal Circulation, Heart Defects, Congenital, Ultrafiltration, Cardiovascular Surgical Procedures.

## Abstract

Cardiopulmonary bypass (CPB) in children presents challenges related to blood volume and surface area of the circuit. Conventional ultrafiltration (CUF) is used to minimize complications, but modified ultrafiltration (MUF) can optimize clinical outcomes. We propose a modification to the CPB circuit, incorporating three luer connectors and a 12 Fr extension tube, allowing for simple and safe MUF implementation. Since 2014, this technique has been applied to approximately 3,500 children weighing < 20 kg, proving to be effective and low-cost. The new configuration does not require additional pumps, facilitates volume replacement, and maintains blood temperature, thereby improving procedural safety. Results indicate that this circuit modification for MUF offers safe and efficient management strategy for pediatric patients, with low risk of complications and potential easy implementation in various cardiovascular surgery centers.

## INTRODUCTION

**Table:** 

Abbreviations, Acronyms & Symbols	
CPB	= Cardiopulmonary bypass
CUF	= Conventional ultrafiltration
MUF	= Modified ultrafiltration
SIRS	= Systemic inflammatory response syndrome

Cardiopulmonary bypass (CPB) in children presents significant challenges regarding circuit-priming volume in relation to blood volume and body surface area^[1]^. These characteristics, along with the interactions of blood with the non-endothelial surfaces of the circuit, influence capillary permeability, leading to increased fluid displacement into the extravascular space, elevated mediators of the systemic inflammatory response syndrome (SIRS), and a higher likelihood of pulmonary and renal injuries^[1,2]^.

Conventional ultrafiltration (CUF) is the classic method to manage these changes during CPB. However, after CPB, modified ultrafiltration (MUF) can be performed. This is an important strategy, particularly in children, as it can potentially optimize CUF by controlling the administered blood volume, reducing edema, and lowering SIRS mediators^[1-3]^.

Understanding and enhancing the use of MUF technique are a significant advancement in the therapeutic approach for these patients, contributing to improved clinical outcomes^[4]^.

**Fig. 1:**
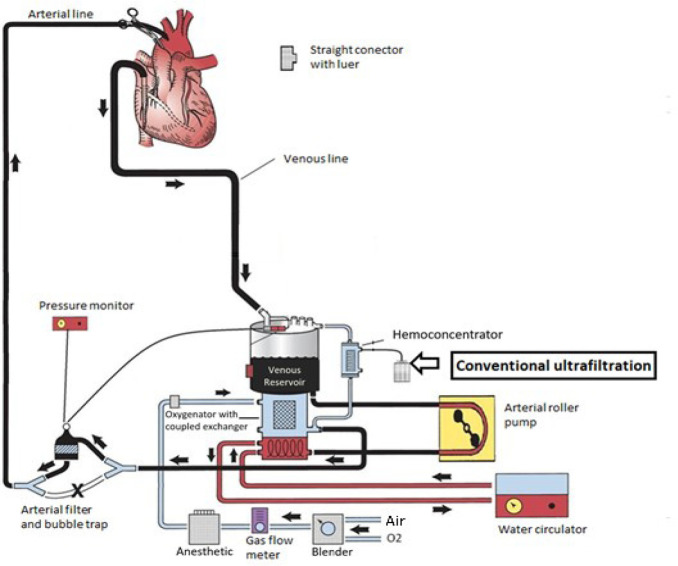
General view of the cardiopulmonary bypass (CPB) circuit in our facility. With this configuration, it is possible to perform conventional ultrafiltration during CPB. The hemoconcentrator connects the outlet of the oxygenator to the inlet of the venous reservoir.

In our setting, the CPB circuit consists of an oxygenator with an integrated heat exchanger, a set of tubing, roller pump, arterial line filter, and hemoconcentrator. This configuration allows for performance of CUF during CPB, with the hemoconcentrator integrated into the circuit, its input line connected to the recirculation outlet of the oxygenator, and the output line directed to the venous reservoir (Figure 1).

We will present a modification to this CPB circuit for the implementation of MUF in a simple, reproducible manner, with low cost and minimal risk of complications.

### Technique

In the CPB circuit, three straight luer connectors and a 12 Fr extension tube, 100 cm in length, equipped with a plastic occluder are incorporated.

**Fig. 2:**
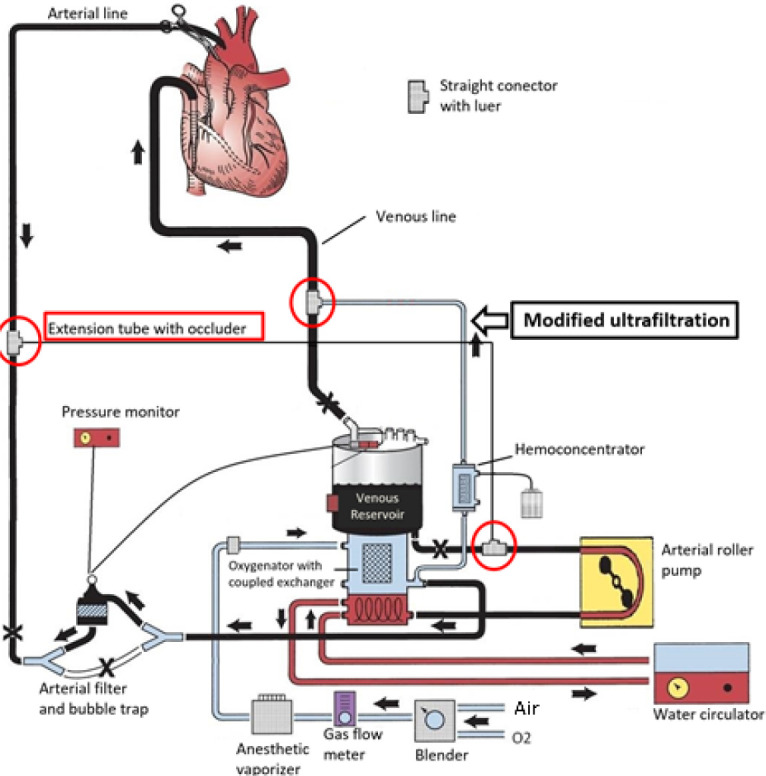
Positioning of three connectors and an extension tube used in the extracorporeal circulation circuit for performing modified ultrafiltration. The connectors can be observed in the arterial line, venous line, and venous reservoir outlet. The extension tube connects the arterial line to the venous reservoir outlet (red).

During assembly of the CPB circuit, one of the straight connectors is inserted into the venous drainage line, immediately before the inlet to the venous reservoir. The other two connectors are positioned just after the outlet of the venous reservoir and the outlet of the arterial blood filter, interconnected by the 12 Fr extension tube (Figure 2).

At the end of CPB, a modification is made to the previously assembled circuit to initiate MUF. The output line of the hemoconcentrator must be disconnected from the venous reservoir and connected to the straight luer connector positioned in the venous drainage line. This line should remain clamped immediately before the inlet to the venous reservoir and the straight luer connector. Additionally, a clamp is inserted between the outlet of the venous reservoir and the straight luer connector that connects to the arterial line.

Thus, the circuit is ready to begin MUF. The occluder on the extension tube is released, and the arterial roller pump is activated to suction blood from the patient's aorta, pushing it to the oxygenator with an integrated heat exchanger and then to the hemoconcentrator, connected to the recirculation outlet of the oxygenator. The blood is filtered and then returned retrogradely to the patient through the venous line.

It is important to emphasize that the flow should be maintained between 100 and 200 milliliters (ml) per minute, and the MUF procedure lasts eight to 12 minutes, provided it does not cause hemodynamic instability. It is crucial to have adequate volume at the end of CPB to perform the procedure without risk of gas embolism or air entry into the system.

Continuous monitoring of mean arterial pressure and near-infrared spectroscopy is conducted to ensure that the patient remains hemodynamically stable during the procedure. If parameters fall below normal for the patient's weight and age, volume infusion is necessary. This should be done by closing the occluder on the extension tube, opening the clamp at the outlet of the venous reservoir, and infusing volume into the patient through the venous drainage line, thereby returning blood retrogradely and stabilizing parameters. Once stable, MUF can be resumed by clamping the outlet of the venous reservoir and releasing the occluder on the extension tube, as previously described (Video 1).

**Video 1:**
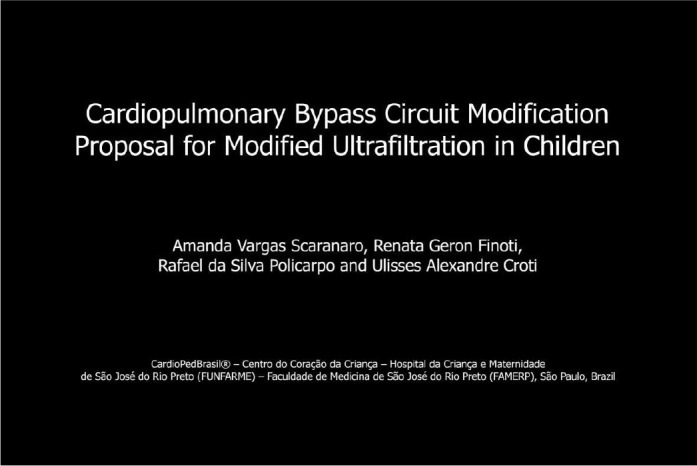
Components and routinely used technique demonstrating the cardiopulmonary bypass circuit modification proposal for modified ultrafiltration in children.

*Link: https://youtu.be/meuX0luGVWk*

## COMMENTS

Since January 2014, we have routinely used this technique in our facility for patients weighing < 20 kg who underwent CPB. Throughout this period, CUF during CPB, followed by MUF after CPB, has been performed in approximately 3,500 patients.

We have observed that the proposed configuration, which is extremely simple, offers significant advantages during the MUF procedure, as it does not require an additional roller pump and consists only of three luer connectors and a single extension tube with an occluder. This allows for easy management of volume replacement, unlike original configurations^[4,5]^.

These components added to the CPB circuit are low-cost and can be easily acquired and adapted to any CPB circuit, regardless of the brand, making it reproducible and usable in any cardiovascular surgery center.

The positioning of the hemoconcentrator at the outlet of the oxygenator recirculation is also an important factor, as it prevents heat loss; the blood first passes through the heat exchanger of the oxygenator, which differs from the configuration typically seen in other CPB circuits. This is particularly important for newborns, who can easily lose heat after CPB^[3]^.

Air entry into the system must be avoided by controlling arterial roller pump flow, which should not exceed 200 ml/min, especially at the beginning of MUF^[5]^. There is always a risk of air bubbles in the hemoconcentrator, and it must be handled properly to ensure any trapped air does not return to the patient. In our experience with the proposed circuit, we have never encountered such complications, leading us to believe that it is a safe and low-risk system.

During MUF, approximately 150 to 250 ml is typically filtered, depending on patient’s weight, remaining volume in the venous reservoir, final volume balance, and hemodynamic stability. Routinely, at the end of MUF, we observe an increase in hematocrit concentration, negative volume balance, improved hemodynamic stability, and edema-free patients. All of this leads to reduced need for blood transfusions in intensive care unit and significant decrease in SIRS^[1,2]^.

## CONCLUSION

The proposed CPB circuit for MUF has proven to be simple, low-cost, and with minimal risk of complications, effectively reducing blood transfusions and allowing for greater hemodynamic stability in the management of pediatric patients.
